# Hospital Meetings, &c.

**Published:** 1900-12-15

**Authors:** 


### HOSPITAL MEETINGS, &c.

### LONDON HOSPITAL.

The customary quarterly meeting of governors of the London Hospital was held on December 4th, the Hon. Sydney Holland presiding.

The report presented, summing up the work done during the year, stated that the demands made on the institution had been heavier than ever. There had been treated during the year no less than 12,035 in-patients, as against 12,365 in the preceding year, and 61,936 out-patients, as against 67,496 in 1899. This heavy work had been met not entirely without inconvenience, as the patients had not been stopped from coming whilst the very extensive improvements were being rapidly pushed forward. Great progress had been made with the front block, and the extension of the receiving room, which was now assuming definite shape, was one which would be highly appreciated and would greatly facilitate the work. The old operating theatres had been given up, and the temporary ones, which answered the requirements of the surgeons, were in use while the new ones were being built. Two floors of the new rearrangement of the centre block were now complete. In regard to the other work, the completion of the post-mortem and pathological department was being rapidly pushed forward, and part of it would probably be in occupation before Christmas. The isolation block being built on the east side of St. Philip's Church was almost completed. The contract for the building of the new out-patient department for a sum of £51,382 had been signed. It was to be completed within a period of twenty-one months from the date of signing, and it was hoped therefore to have it done by August, 1902. The demolition of the old buildings was already in progress. A smaller but very important re-arrangement of the building had been made in regard to the nurses' home kitchen. The old kitchen had become too small for the more extended work, and it had therefore been enlarged with very satisfactory results. The general work of the hospital had progressed, and the attention of the governors was particularly called to the continued success of the light department for the treatment of lupus. It was just six months since, by the generous assistance of the Princess of Wales, the department was first opened. On September 3rd the second light was started, and there were now being treated every morning at least 24 cases during a period of three hours. In the afternoon there were received 8 paying patients per hour. These latter helped to meet the heavy expense entailed. Since the department was opened 125 patients had been treated, 48 being cured, and 77 were still under treatment. There was a waiting list of 138 patients anxious to be treated as soon as arrangements could be made to receive them.

The wider development of the radiographic and photographic department had had to be faced; the whole of that work had now been fully reorganised to meet present demands, and the work was now being most efficiently done. To meet the increasing demands in the special departments the committee had decided on the appointment of a paid ophthalmic clinical assistant. The numbers of patients attending the eye department showed a considerable increase, and it was found by the surgeons in charge quite impossible to get through the work in the two mornings without such assistance as had been given. The committee had also prepared for the carrying-out of the work in the new post-mortem department, and had appointed Dr. Bulloch demonstrator of pathological histology, with supervision of the work to be carried on in the post-mortem department.

There had, unfortunately, been a certain amount of sickness amongst the nursing staff during the past quarter, no less than three of the private nursing staff having contracted enteric fever whilst at work away from the hospital. The committee had also to report, with the deepest regret, the death, from pneumonia, of Miss Illingworth, one of the probationers, after a very short illness. Much pleasure had been given to the nurses during the summer and autumn season by the kindness of Mr. Hughes, of the London and Tilbury Lighterage Company, no less than 840 of the nurses having enjoyed the river trips arranged by him, and which afforded an ideal recreation for nurses after a night or day on duty.

In conformity with the wishes of the executors under the will of the late Herman de Stern, a site had been purchased for £900 by the trustees of the hospital for the erection of the Herman de Stern Convalescent Home at Felixstowe. A site had also been bought for £600, adjoining the other, with a view to being ready for probable developments of this very important branch of hospital work. The estate of the greatest benefactor the hospital had ever had—Professor David Hughes—was gradually being wound up, and now Mr. John Henry Buxton, the treasurer of the hospital, had agreed to become trustee under the terms of the will. To meet the expenses of the alterations, and in accordance with the conditions under which the grant from the Prince of Wales's Fund was made, one of the investments—Canada Three per Cent.—had been sold out, a good price being realised. Through failing health Mr. Rampley, the surgery beadle, had been obliged to give up the duty he had so ably performed for so many years, and the reorganisation of the department was now being fully considered. It is noteworthy that Mr. Rampley had been present at nearly 40,000 major operations in the theatres of the hospital. It had also been reported to the governors that Mr. Robertson, late chief dispenser, whose pension the governors considered it necessary to stop, brought an action for the continuance of that pension. On the case proceeding, however, he decided to withdraw unconditionally, throwing himself on the mercy of the governors. In view of the long term of his service and the fact that he was himself very poor, the committee had decided to make him an allowance of £1 per week during their pleasure.

In moving the adoption of the report the chairman referred to the circumstances under which one of the donations—£2,500 to endow two beds and a cot—was made. Mr. R. W Payne, their house physician and a scholar of the college whose death was really due to devotion to duty, blood-poisoning having been contracted in the course of his work, had on his death-bed expressed to his father his desire that out of his own property this endowment might be made. The governors would be glad to know of the continued satisfaction of the committee with the Lupus cure. The only word which would at all describe the results obtained was "miraculous." Already forty-eight poor people, who previously had not been able to show their faces owing to the hideous disfigurement of the disease, had been absolutely cured, though it was only six months since the installation of the light cure. Replying to a suggestion that bearers of letters from societies which subscribed to the hospital should on that account be let off the payment asked from out-patients, Mr. Holland pointed out that the sum to be paid was only 3*d.*, toward the cost of the medicine supplied—the consultation, which would cost him a couple of guineas, being thrown in free. In every case where a person was too poor to afford it he was let off; and he thought that those who grumbled at the trifle would think nothing of getting themselves a pint of beer as they came along. The benefits of the hospital were no longer restricted to those who had letters, and they were really doing much more for the societies than ever before. An in-patient, for instance, might stay here for three months absolutely without charge. And, finally, he pointed out that on the day the governors passed such a resolution as that suggested, the cheque for £25,000 given for the extension of the out-patient department must be returned, it being a condition of the donor that letters should not be required.

The report was adopted; the re-election of the vice-presidents, with the addition of the name of Mr. Carr Gomm; of the committee, and of the staff, with other formal business, was voted, and the meeting terminated with thanks to the chairman.

